# Mental health and quality of life outcomes after CABG in a South Asian country: a prospective study

**DOI:** 10.1186/s13019-026-04116-7

**Published:** 2026-05-05

**Authors:** Asma Altaf Hussain Merchant, Mohammad Umair Khan, Ebadullah Shahood Ahmed, Areesha Ahmer, Mustafa Ali Khan, Malik Muhammad Hamza Khan, Mohammad bin Pervez, Shahid Sami, Syed Shahabuddin, Ashar Khan, Saulat Hasnain Fatimi

**Affiliations:** 1 Medical College, Aga Khan University, Karachi, Pakistan; 2 Department of Cardiothoracic Surgery, Aga Khan University, Karachi, Pakistan; 3 Queensland ADHD and Neurosciences Clinic, Queensland, Australia

**Keywords:** Quality of life, Coronary artery bypass grafting, Depression, Anxiety, Ischemic Heart Disease

## Abstract

**Background:**

Coronary artery bypass grafting (CABG) is a proven life-saving intervention for ischemic heart disease (IHD). However, literature on its post-surgical quality of life (QoL) and mental health is limited in resource-constrained settings, where IHD burden remains high. This study determined changes in depression, anxiety, and QoL pre-operatively to 1- and 6-month post-CABG in a South Asian low- and middle-income country (LMIC).

**Methods:**

This prospective study was conducted at a large private hospital in Pakistan (February 2022-March 2023). Data were collected pre-operatively, 1-month, and 6-month post-CABG. Beck Depression Inventory II (BDI-II), Beck Anxiety Inventory (BAI), and 36-Item Short Form Health Survey (SF-36) were used to assess depression, anxiety, and QoL, respectively. Pre-operative scores were compared with 1-month and 6-month post-operative scores using paired t-tests, with p-value < 0.05 considered significant.

**Results:**

The mean age of 82 patients with complete pre-operative data was 58.48 ± 10.07 years. 1- and 6-month follow-up rates were 63% and 53.7%, respectively. No significant differences were noted for depression, but mean anxiety scores reduced preoperatively to 6-month postoperatively (7.48 ± 8.44 to 4.39 ± 7.25, p-value:0.0216). 6-month postoperative results demonstrated significant improvements from preoperative scores in physical functioning (57.39 to 81.36), role limitations due to physical health (32.39 to 69.89), role limitations due to emotional problems (58.33 to 84.09), and general health perceptions (71.36 to 81.36).

**Conclusion:**

This study showed improvement in anxiety and QoL 6 months after CABG. However, depression scores remained unchanged. Interventions like pre-operative counselling, post-operative cardiac rehabilitation, and long-term psychiatric evaluation are critical to support sustained recovery post-CABG.

## Key learning points

**What is already known:**

- Coronary artery bypass grafting (CABG) improves morbidity and mortality in patients with ischemic heart disease (IHD). However, its impact on the post-surgical quality of life (QoL) and mental health parameters remains underexplored, especially in low- and middle-income countries (LMICs).
- Studies from high-income countries have shown that patients undergoing CABG often experience depression and anxiety pre- and post-surgery, requiring long-term psychological monitoring and interventions.

**What this study adds:**

- This prospective study provides evidence from a South Asian LMIC that demonstrates improvements in anxiety and multiple QoL domains 6 months after CABG, without significant changes in depression from preoperative to postoperative scores.
- Findings from this study underscore the importance of ongoing psychological monitoring beyond the 6 months after CABG to account for long-term changes in patients’ QoL and mental health.

## Background

Ischemic heart disease (IHD) continues to be one of the leading causes of mortality and morbidity globally, accounting for 8.99 million deaths in 2021 [1, 2]. Low- and middle-income countries (LMICs) face a disproportionate burden of IHD, with 94.2% of IHD-associated deaths attributed to modifiable risk factors [3]. Pakistan, a South Asian LMIC, has an age-standardized incidence rate of 918.18 per 100,000 cardiovascular diseases (CVDs), surpassing the global rate of 684.33 per 100,000 [4]. Consequently, the country faces a high mortality rate related to IHD, with approximately 120,000 deaths secondary to dietary risks and 33,000 deaths due to high body mass index (BMI) [5].

The gold standard for treatment of advanced IHD is coronary artery bypass graft (CABG) surgery, which also leads to improvements in the patients’ symptoms and quality of life [6]. Traditionally, medical interventions like CABG have been assessed through morbidity and mortality rates, indicating risks such as stroke, infection, hemorrhage, myocardial infarction, or death [7]. While these outcomes are crucial and measurable, they alone cannot adequately represent the entire picture. Though the primary medical priority is mortality benefit, symptomatic relief and quality of life also rank high on the patients’ agenda. Hence, increasing emphasis is being laid on the importance of evaluating more subjective measures such as changes in mental health and Health-Related Quality of Life (HRQoL) [8, 9].

Literature suggests a reduction in depression and anxiety, and improvement in quality of life (QoL) after CABG globally [10–12]. However, the extent of these benefits can vary among various patient populations. Given the scarcity of associated evidence from Pakistan despite the extensive IHD burden, this study aimed to determine the change in depression, anxiety, and QoL parameters from the pre-operative period to 1- and 6-months post-CABG.

## Methodology

### Study design, duration, and setting

This prospective cohort study was conducted at the Aga Khan University Hospital (AKUH), one of the largest private hospitals in Karachi, Pakistan, with a dedicated coronary care unit (CCU) and a 10-bedded cardiac intensive care unit (CICU). Patients were enrolled in the study from February 2022 to August 2022 and followed for six months post-operatively.

### Participant enrollment

Assuming a moderate effect size of 0.5, a two-tailed significance level of 0.05, and a desired power of 90%, the minimum required sample size was calculated to be 44 to detect a meaningful change in Beck Depression Inventory II (BDI-II), Beck Anxiety Inventory (BAI), and 36-Item Short Form Health Survey (SF-36) components measured pre-operatively, at 1- and 6-month post-operatively. G*Power software Version 3.1.9.7 was used for this analysis [13]. Assuming a follow-up of 50% at a 6-month interval [14], the sample size was inflated by 22 participants. However, during 1-month follow-up, extensive loss-to-follow-up was noticed, which led to enrolling more participants using convenience sampling to ensure adequate power at the end of the study.

Participants were enrolled using non-probability consecutive sampling, where all eligible patients who presented to the cardiothoracic surgery department during the study enrollment period for an elective CABG were asked to participate. Patients aged less than 18, undergoing emergent CABG or an intervention other than isolated CABG, lacking sufficient comprehension skills to provide consent or fill a self-assessment form, pregnant patients, those with dementia, intellectual disability, or other diagnosed cognitive deficit were not eligible for participation in the study. Additionally, patients already using antidepressant/anti-anxiety therapy (pharmacological and non-pharmacological) or who had terminated their depression/anxiety treatment two months before enrollment in the study were excluded. Any patient who died during the surgery due to intraoperative complications was also excluded.

### Consent

This study was carried out in accordance with the ethical principles of the Declaration of Helsinki. All eligible patients were approached and invited to participate in the study one day before their surgery, after their admission to the hospital. Prior to patient enrollment, all research team members involved in data collection underwent standardized training sessions, which included detailed instructions on administering the informed consent, explaining the study procedures, and accurately filling out the study forms.

A written informed consent was administered in English/Urdu based on the patient’s preference. Additionally, the study’s aims, objectives, risks, benefits, and follow-up procedure were explained to the participants by a member of the research team. To ensure confidentiality of the participants, each patient was given a de-identified subject ID, which was used to collect follow-up data. Identifiers correlating to each subject ID were kept in a password-protected file accessible only to the primary study investigator.

### Data collection

After ensuring the patient’s understanding and receiving consent, the research team member filled in a proforma for each enrolled patient regarding their demographics, comorbidities, and pre-operative medications, which were later completed with pre- and peri-operative details, including pre-operative left ventricular ejection fraction (LVEF), total operative time, extra-corporeal circulation (ECC) time, and aortic cross-clamp time (XCT).

The patients were asked to fill out three surveys pre-operatively after providing consent: (1) BDI-II, (2) BAI, and (3) SF-36. The BDI-II is a 21-item, self-reporting, well-validated questionnaire, with an internal mean consistency of 0.86, that measures the symptoms and severity of depression [15, 16]. The BAI is a 21-item, self-reporting measure to indicate the severity of anxiety, with a high internal consistency (Cronbach’s alpha: 0.92) [17]. SF-36 is a self-administered, 36-item survey that covers eight domains (physical functioning, social functioning, role limitations due to physical health, role limitations due to emotional health, general mental health, vitality (energy and fatigue), bodily pain, and general health perceptions) [18]. Both English and Urdu versions have been shown to have high reliability with a Cronbach’s alpha of > 0.85 and 0.73–0.90, respectively [19, 20].

Patients were followed up after a period of 1- and 6-month post-operatively via telephone and during in-person clinic visits, where they were asked to fill in the three surveys again. A standardized script was utilized to ensure consistency in questions. The telephone follow-ups were done in a private and quiet environment to avoid interruptions. The follow-up calls were initiated at exactly 1- and 6-month post-operative dates, where the participant was called up to a maximum of three times with 7-day intervals. If the participant did not respond within this time, they were marked as lost to follow-up. Data collected through telephonic interviews/in-person visits were entered on a password-protected sheet, accessible only to the study members collecting and analyzing data.

### Statistical analysis

Data analysis was performed using StataCorp. 2015. Stata Statistical Software: Release 14. College Station, TX: StataCorp LP. For continuous variables, the mean and standard deviation were calculated, while frequencies and percentages were calculated for categorical variables. Paired t-tests were used to determine the mean scores of BDI-II, BAI, and SF-36 components, comparing the change between pre-operative scores, 1-month and 6-month scores. A p-value of < 0.05 was considered statistically significant.

## Results

A total of 87 patients were enrolled in the study, but due to missing pre-operative data, 5 records were excluded. Analysis was conducted for 82 records. The mean age of participants was 58.48 ± 10.07 years, where 72 (87.80%) patients were males, and 10 (12.20%) patients were females. The mean BMI was 28.07 ± 4.92 kg/m^2^. Most participants presented with New York Heart Association (NYHA) classification of I (*n* = 36, 43.90%), followed by 25 (30.49%) patients with class II, 18 (21.95%) with class III, and 3 (3.66%) patients with class IV. Most of the participants were non-smokers (*n* = 37, 42.53%), while 9 (10.98%) patients were current smokers. Hypertension (73.17%), diabetes mellitus (70.73%), and dyslipidemia (68.29%) were the most common comorbidities.

LVEF was not recorded within the hospital charts for 3 patients, resulting in a mean LVEF of 49.45% ± 11.09% for 79 patients. While CABG was the first intervention for IHD in 69 (84.15%) patients, 13 (15.85%) patients underwent a CABG following a previously performed percutaneous coronary intervention (PCI). Mean length of stay (LOS) was 7.01 ± 1.72 days. Table 1 provides further demographic and perioperative details.

**Table 1:** Demographic and Peri-operative Details of Participants

Variable	*N* = 82 *n* (%)
Age (Mean ± SD)	58.48 ± 10.07
**Sex** Male Female	72 (87.80) 10 (12.20)
**NYHA Class** I II III IV	36 (43.90) 25 (30.49) 18 (21.95) 3 (3.66)
**Smoking Status** Smoker Ex-smoker Non-smoker	9 (10.98) 36 (43.90) 37 (42.53)
**Comorbidities** Hypertension Diabetes Mellitus Dyslipidemia CHF Peripheral vascular disease Cerebral vascular disease Chronic kidney disease Past psychiatric history	60 (73.17) 58 (70.73) 56 (68.29) 1 (1.22) 0 (0.00) 3 (3.66) 6 (7.32) 3 (3.70)
**Any Substance Use** Yes No	35 (40.23) 47 (59.77)
**Days to Diagnosed MI** 3–7 das 8–21 days > 21 days Never	5 (6.10) 15 (18.29) 13 (15.85) 49 (59.76)
Left Ventricular Ejection Fraction (%) (Mean ± SD) (*N* = 79)	49.45 ± 11.09
**Procedure History** First CV Surgery History of PCI	69 (84.15) 13 (15.85)
**Perioperative Details**	
Total Operative Time (minutes) (Mean ± SD)	260.27 ± 56.00
Extracorporeal circulation time (minutes) (Mean ± SD)	107.71 ± 25.34
Aortic cross clamp time (minutes) (Mean ± SD)	61.96 ± 17.72
CICU stay (hours) (Mean ± SD)	34.89 ± 26.20
Length of hospital stay (days) (Mean ± SD)	7.01 ± 1.72

Approximately 63% (*n* = 52) of patients completed 1-month follow-up postoperatively, while 53.7% (*n* = 44) of patients completed the follow-up at 6-month postoperatively. As shown in Table 2, no significant differences were seen in anxiety and depression scores at one month postoperatively compared to preoperative scores; however, mean anxiety scores significantly reduced from the preoperative period to 6-month postoperatively (7.48 ± 8.44 to 4.39 ± 7.25, p-value: 0.0216).

**Table 2:** Pre-operative and post-operative scores for BAI, BDI, and SF-36 components

Variable	Pre-operative Score *N* = 82	Scores for patients who completed 1-month follow-up *N* = 52			Scores for patients who completed 6-month follow-up *N* = 44		
	Mean ± SD	Pre-Op Mean ± SD	Post-Op Mean ± SD	*p*-value	Pre-Op Mean ± SD	Post-Op Mean ± SD	*p*-value
BAI	7.62 ± 8.41	7.90 ± 8.30	6.54 ± 7.17	0.3078	7.48 ± 8.44	4.39 ± 7.25	**0.0216**
BDI	5.61 ± 7.06	5.48 ± 6.61	6.77 ± 6.32	0.2209	4.14 ± 5.57	3.84 ± 4.76	0.7822
Physical Function	56.40 ± 29.71	59.90 ± 28.74	34.33 ± 20.24	**< 0.0001**	57.39 ± 28.64	81.36 ± 22.60	**< 0.0001**
Role Limitations due to Physical Health	33.23 ± 38.10	37.50 ± 39.14	24.04 ± 28.42	**0.0489**	32.39 ± 34.35	69.89 ± 41.27	**< 0.0001**
Role Limitations due to Emotional Problems	58.54 ± 44.31	60.90 ± 42.12	68.59 ± 45.45	0.3008	58.33 ± 43.21	84.09 ± 27.36	**0.0006**
Vitality (energy and fatigue)	71.34 ± 21.11	72.40 ± 19.47	70.19 ± 21.17	0.4804	72.95 ± 21.82	66.36 ± 20.70	0.0830
General Mental Health	83.95 ± 18.89	85.15 ± 16.00	86.69 ± 18.01	0.5955	86.64 ± 17.51	89.18 ± 12.06	0.3854
Social Functioning	74.54 ± 27.46	73.08 ± 28.37	74.76 ± 31.55	0.7592	79.55 ± 26.84	80.97 ± 24.93	0.7783
Bodily Pain	68.63 ± 26.09	70.10 ± 26.82	66.59 ± 27.66	0.5257	73.92 ± 21.59	42.95 ± 22.61	0.0564
General Health Perceptions	69.63 ± 22.98	69.42 ± 22.87	78.85 ± 20.36	**0.0026**	71.36 ± 22.68	81.36 ± 15.19	**0.0021**

Paired t-tests for comparison of means; bold values indicate statistically significant difference between the mean scores of the measured parameter pre-operatively and post-operatively (p-value < 0.05 considered statistically significant)

No statistically significant differences were seen among the QoL components of SF-36 from pre-operative period to 1-month postoperatively, except for the extent of physical functioning which worsened significantly (59.90 to 34.33; p-value < 0.0001), performing chores due to physical health became further limited (37.50 to 24.04; p-value: 0.0489), and general health was seen to be better perceived (69.42 to 78.85; p-value: 0.0006). Compared to the pre-operative period, significant improvements were noted 6-month post-operatively in physical functioning (57.39 to 81.36; p-value < 0.0001), role limitations due to physical health (32.39 to 69.89; p-value < 0.0001), role limitations due to emotional problems (58.33 to 84.09; p-value: 0.0006), and general health perceptions (71.36 to 81.36; p-value: 0.0021).

The results also demonstrated a decline in the vitality of the patients 6-month post-operatively compared to before undergoing CABG, but this was not considered statistically significant (72.95 to 66.36; p-value: 0.0830). Changes in general mental health, social functioning, and bodily pain domains were not statistically significant.

## Discussion

This prospective study followed CABG patients 1- and 6-months post-operatively to determine the changes in depression, anxiety, and QoL, compared to their pre-operative state. Our results demonstrate a decline in the physical function by the patients 1-month post-operatively, but a significant reduction in anxiety and improvement in the overall QoL 6-month post-operatively, with no significant difference in depression.

While the maximum score that can be obtained on BAI is 63, the mean score among our initial cohort of patients pre-operatively was 7.62, falling under the category of minimal-mild anxiety [21]. Similar findings have been reported from a study in Turkey, where pre-operative BAI score was measured as 11.28 ± 7.28 [22]. However, this is a striking contrast to Ramesh et al.’s results, where the patients had considerable anxiety pre-operatively measured through the *State Trait Anxiety Inventory* (STAI; mean score: 51.03 ± 10.50) [23]. Anxiety itself is a common response to environmental and psychosocial stressors, and would be especially observed in response to a life-changing event like CABG surgery. Pre-operative anxiety is mainly linked to the uncertainty and complexity of the surgery, and can present with symptoms that overlap with the patient’s cardiac symptoms, potentially worsening their status and complicating their assessment [24]. Pre-operative education and counselling of the patients has been shown to reduce anxiety levels among CABG patients [25]. AKUH has a rigorous pre-education practice for CABG patients, where the primary surgeon ensures patients’ understanding of the need for the surgery, followed by constant guidance of the pre-operative procedures by the postgraduate trainees and nursing staff [26]. This could be one of the reasons for low anxiety levels among the participants in this study. Additionally, the female gender has been shown to have higher rates of pre-operative anxiety [23, 27]. Since our cohort only consisted of 12.20% of females, the anxiety scores could have been skewed towards the lower side. Comparison of the pre-operative BAI scores with 6-month post-operative scores showed a statistically significant decline of more than 3 points. The limited literature on long-term anxiety levels suggests congruent findings from a study in Poland, which demonstrated a significant reduction of 7.6 points in STAI from pre-operative to 6-month postoperative interval (p-value < 0.05) [28]. Another study from Poland also indicated a significant decrease in anxiety scores at 3-month and 8-year post-operative intervals [29].

It was interesting to note that while BDI scores measuring depression increased after one month of surgery and decreased 6-month postoperatively, these changes were not significant. Kustrzycki et al. concluded similar findings, where the decrease in BDI scores was not significant at 3-month and 8-year intervals post-operatively [29]. In contrast, a study from Iran showed a decrease of 2.1 using the Hamilton Depression Scale to be statistically significant [30]. In a meta-analysis of 39 studies, it was further noted that measurement of depression in CABG patients using a dichotomous scale showed significant reductions as compared to those measured on continuous scales [10]. A few reasons for the low levels of anxiety and depression among our participants could allude to the protective role of religion [31, 32], since Pakistan is a predominantly Muslim country, with most of the population being Muslims. A higher level of social support through joint and extended families within the country also offers greater emotional and social support [33], which further minimizes postoperative anxiety and depression [34, 35]. Another reason contributing to this finding could also be masked depression among males, who generally cope with depression by omitting their symptoms and attributing showing of such symptoms as shameful [36]. Given that this study had 87.80% of males, it could result in a low depression prevalence owing to males not specifying their symptoms. This finding is further congruent with both general patterns of depression [37], and those associated with CABG globally [8].

While our results showed increased limitation in physical functioning 1-month after CABG, a study from Croatia indicated improvements in all domains of SF-36, except for social functioning [38]. Post-operative symptoms such as fatigue, pain, and dyspnea can limit physical functioning post-CABG [39]. These initial limitations may also be associated with features of somatic symptom and related disorders, as patients can become preoccupied with their symptoms. This preoccupation may lead to a subconscious exaggeration of pain and other discomforts, potentially delaying recovery and functional improvement. Additionally, sternal precautions often hinder patients from fully returning to their pre-operative functionality [39, 40], which could be explained by the worsening of the physical scores in our study.

Minimal clinically important differences (MCIDs) for SF-36 subscales have not been well established in CABG populations. In studies of other clinical populations, including rheumatoid arthritis, changes of approximately 3, 5, or 10 points on individual subscales and 2.5–5 points on the physical and mental component summaries have been cited as clinically meaningful [41]. Therefore, these domains were used to establish whether the statistical improvements also translated to clinical improvements. Using these benchmarks, our results concluded significant clinical improvement in 6 months post-operatively in physical functioning, role limitations due to physical health, role limitations due to emotional problems, and general health perceptions compared to pre-operative scores. This suggests greater improvement in physical domains of the life of patients, compared to the emotional and mental ones, since CABG allows strengthening of one’s ability to perform activities of daily living. Similar findings have been presented in the literature, where a study from India showed a higher increase in physical components of SF-36 than the mental components after 3 months of surgery (Physical: 34.57–55.34, p-value < 0.01; Mental: 43.53–53.83, p-value < 0.05) [42]. Similarly, a systematic review assessing studies with SF-36 highlighted greater physical improvements after CABG, ranging from a 2.2 to 8.2-point increase, whereas the mental aspect of QoL improved by 0.3 to 3.6 points [43]. Another meta-analysis of 18 randomized controlled trials (RCTs) did not show significant improvement in the mental component of SF-36 1-year after CABG [12]. While psychosocial therapy and cognitive behavioral therapy can improve the mental health of CABG patients, such interventions and associated literature are limited [44, 45]. Further studies in the context of the South Asian population are required to determine the impact of such interventions on the mental component of the QoL among CABG patients.

This is one of the first prospective studies of its kind from Pakistan to determine long-term changes in depression, anxiety, and QoL parameters among CABG patients. The use of validated tools strengthens the reliability of our findings. However, there are some limitations. The patients were included from a single center and were predominantly males, which could limit generalizability to other settings. A significant limitation was the high loss-to-follow-up. Such attrition is common in longitudinal studies within LMICs like Pakistan. Lannon et al. indicated that even with phone access, there is a significant loss in LMICs for post-cardiac surgery follow-ups, owing to transient phone subscriptions and employment-dependent phone access [46]. Other factors contributing to the attrition may include socioeconomic constraints limiting time for calls, difficulties in maintaining updated contact information amidst population mobility, and patients discontinuing participation due to perceived symptom resolution post-surgery. There is also a possibility of attrition bias, where patients experiencing better psychological recovery may have been more likely to respond to follow-up assessments. This could have influenced the observed outcomes by limiting the representativeness of the follow-up sample and potentially affecting the interpretation of longitudinal changes in mental health and quality of life. Scheduling in-person follow-ups and ensuring that the same team members who collected pre-operative data follow up with their patients might have reduced the loss-to-follow-up rate and further strengthened the findings. The analysis relied primarily on paired t-tests to assess within-subject changes over time. This analytical technique was appropriate for our study to evaluate longitudinal trends in mental health outcomes, but it did not adjust for potential confounders such as age, sex, and baseline NYHA class. Future studies with larger cohorts could use multivariable or mixed-effects models to account for these variables and provide more robust estimates. Lastly, the surveys and follow-ups were mainly carried out by medical students rather than psychiatrists, who would generally be more experienced in utilizing the questionnaires; such psychiatrists are rather few in number and difficult to involve in localized research, particularly in LMICs like Pakistan [47].

This study’s findings have implications for further investigations within the South Asian region and beyond to determine both short- and long-term changes in psychological parameters of CABG patients. Determination of such changes can provide evidence to advocate for pre- and post-operative interventions that could reduce anxiety and depression among the patients and improve their long-term QoL, where they could return to their healthy pre-operative state. Longer follow-up times and the conduction of mixed-methods, multi-center studies can provide granular data that can be leveraged by healthcare professionals to ensure patients return to their pre-operative health after undergoing a surgical intervention like CABG.

## Conclusion

It is vital to prioritize both physical and mental well-being among patients undergoing CABG surgery. This study exhibits a significant reduction in anxiety and improvement in components of QoL, including physical functioning, role limitations due to physical health and emotion problems, and general health perception 6 months post-operation. Further studies are required to indicate contextual barriers to improved mental and emotional health post-CABG, which could be translated into actionable strategies for the betterment of patients’ lives in the long term. Interventions such as pre-operative counselling, post-operative cardiac rehabilitation, and long-term follow-ups for psychiatric evaluation, such as at 12 to 24 months post-operative period, are critical to sustaining improvements in patients’ overall health after CABG.

## Data Availability

The data that support the findings of this study are available from the corresponding author upon reasonable request.
